# Leukocyte populations and their cell adhesion molecules expression in newborn dromedary camel calves

**DOI:** 10.14202/vetworld.2020.1863-1869

**Published:** 2020-09-12

**Authors:** Muaadh M. Gaashan, Abdullah I. A. Al-Mubarak, Jamal Hussen

**Affiliations:** Department of Microbiology, College of Veterinary Medicine, King Faisal University, Al-Ahsa, Saudi Arabia

**Keywords:** adhesion molecules, flow cytometry, immunophenotype, leukocytes, newborn camel calf

## Abstract

**Background and Aim:**

Different properties of the newborn immune system have been characterized in many species. For the newborn camel calf, however, the phenotype and composition of blood leukocytes have so far not been evaluated. The current study aimed to analyze the distribution of leukocyte subpopulations and their expression pattern of cell adhesion molecules in newborn and adult dromedary camels.

**Materials and Methods:**

Blood samples were collected from 17 newborn camel calves and 32 adult camels. For each sample, total leukocytes were separated and analyzed for their composition and cell adhesion molecules expression by flow cytometry.

**Results:**

In comparison to adult camels, newborn camel calves had higher leukocyte numbers and higher numbers of neutrophils, monocytes, and lymphocytes but lower numbers of eosinophils in their blood. Among the lymphocyte populations in calves, the fractions of B cells and γ^δ^ T cells were elevated when compared to adults, whereas CD4-positive T cells were reduced. The comparison between camel calves and adult camels revealed significantly lower expression of the cell adhesion molecules CD11a, CD11b, and CD18 on granulocytes, monocytes, and lymphocytes in calves.

**Conclusion:**

Newborn camel calves show a distinct composition and phenotype pattern of blood leukocytes when compared to adult camels. The observed rise in many leukocyte populations in calf blood may be due to reduced migratory activity in calf leukocyte populations.

## Introduction

Newborn camel calves show high susceptibility to infectious diseases, which are responsible for increased mortality rates during the 1^st^ week after birth [1-7]. The immune responses of the newborn are not as fully mature as the immune responses in adults [8]; age-related changes in different aspects of the innate and adaptive immune responses have been described for different species [9,10].

Blood leukocytes, which contain a reservoir of cells of both innate and adaptive immunity, provide an easily accessible sample source for studying the immune system. Leukocyte composition and immunophenotype are influenced by different physiologic [11] and pathologic factors [12]. According to studies conducted on other species, the composition and phenotype of blood leukocytes are significantly affected by an animal’s age [9,13,14]. With advanced age, a decrease in lymphocyte counts and an increase in neutrophils counts have been reported. In addition, studies have found major differences in the distribution of cell subsets among the whole monocyte and lymphocyte populations between young and adult animals [15,16].

Leukocyte extravasation is a complex process mediated by the interaction of several adhesion molecules on the surface of leukocytes with molecules expressed on endothelial cells of blood vessels [17-20]. Mac-1 (a dimer of CD11b and CD18) is mainly expressed on myeloid cells, such as granulocytes and monocytes [21]. The lymphocyte function antigen-1, which is made by the dimerization of CD11a and CD18, is expressed on all leukocyte populations [19]. Age-related changes in the expression pattern of human leukocyte adhesion molecules have been reported in different studies [9,22-24].

As the composition and phenotype of blood leukocytes in newborn camel calves have not been widely studied, the current study aimed to analyze the distribution of leukocyte populations and evaluate their expression pattern of cell adhesion molecules in newborn and adult dromedary camels.

## Materials and Methods

### Ethical approval

The project underwent ethical review and was approved by the Ethics Committee at King Faisal University, Saudi Arabia (KFU-REC/2019-10-01). The care and use of experimental animals complied with local animal welfare laws, guidelines, and policies.

### Animals and blood sampling

The study was conducted between October 2019 and April 2020 at the Camel Research Center of King Faisal University in Saudi Arabia. Forty-nine camels (*Camelus dromedarius*) were included in the study: 17 newborn camel calves (aged between 7 and 20 days) and 32 adults (aged between 6 and 12 years). All were apparently healthy animals. Blood samples were collected by venipuncture of the vena jugularis externa into Vacutainer tubes containing EDTA (Becton Dickinson, Heidelberg, Germany).

### Cell separation and leukocyte counting

**Figure-1:**
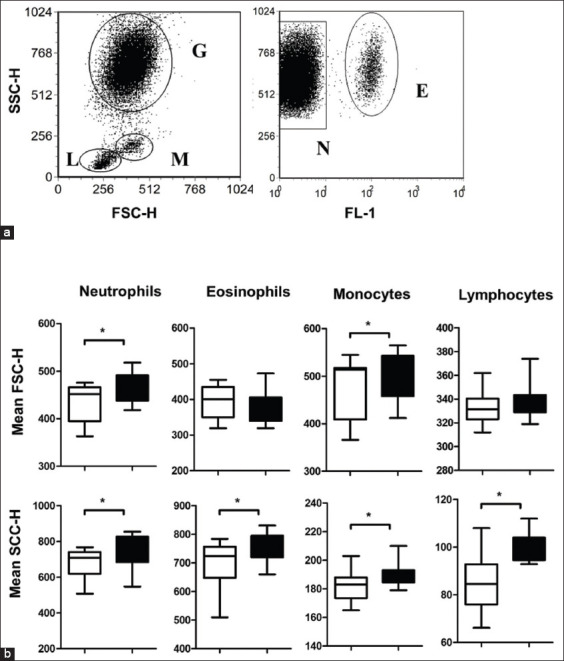
Gating strategy for the identification of the main leukocyte populations in the peripheral blood of newborn camel calves. (a) In a side scatter (SSC)/forward scatter (FSC) dot plot, camel granulocytes (G), monocytes (M), and lymphocytes (L) were gated according to their FSC and SSC characteristics. After setting a gate on granulocytes, eosinophils (E) and neutrophils (N) were identified according to their different autofluorescence intensities in the FL1 fluorescence channel. (b) For each leukocyte subpopulation, the mean SSC and FSC values were calculated and presented comparatively for newborn and adults (*p<0.05).

Separation of blood leukocytes was done after the hypotonic lysis of erythrocytes [25]. Briefly, the collected blood samples (diluted 1 to 1 in phosphate-buffered solution [PBS]) were centrifuged (800× *g*) for 20 min at 10°C without a break. After removing the supernatant (plasma), the erythrocytes were lysed by adding distilled water for 20 s, and the tonicity was restored by adding the same volume of double concentrated (2×) PBS. This was repeated until the complete lysis of erythrocytes. The remaining cell pellet was washed 3 times in PBS (centrifuged at 500× *g*, 250× *g*, and 100× *g*), each for 10 min. Separated cells were finally suspended in membrane immunofluorescence (MIF) buffer (PBS containing bovine serum albumin [5 g/L] and NaN_3_ [0.1 g/L]) at 6×10^6^ cells/mL. The viability of separated leukocytes (>95%) was evaluated by dye exclusion (propidium iodide; 2 μg/mL, Calbiochem, Germany). For absolute counting of total leukocytes, blood was diluted 1:2 in PBS and was then mixed with Türk Solution (Sigma-Aldrich, USA), and the mixture was poured onto the Neubauer counting chamber [26]. Cells were counted in four large squares under a microscope, and total leukocyte count was calculated (cell/μL blood). After the flow cytometric estimation of the percentages of different leukocyte populations (Figure-1a), the absolute cell number of neutrophils, eosinophils, monocytes, and lymphocytes was calculated relative to the total number of leukocytes.

### Monoclonal antibodies

Monoclonal antibodies used in this study are listed in Table-1.

**Table-1:** List of primary and secondary antibodies.

Antigen	Antibody clone	Labeling	Source	Isotype
MHCII	H58A	Unlabeled	WSU	Mouse IgG2a
Bovine CD4	GC50A1	Unlabeled	WSU	Mouse IgM
Bovine WC1	BAQ128A	Unlabeled	WSU	Mouse IgG1
CD11a	G43-25B	PE	BD	Mouse IgG2a
CD11b	ICRF44	PE-Cy7	BD	Mouse IgG1
CD18	6.7	FITC	BD	Mouse IgG1
Mouse IgM	Polyclonal	APC	Thermo Fisher	Goat IgG
Mouse IgG1	Polyclonal	FITC	Thermo Fisher	Goat IgG
Mouse IgG2a	Polyclonal	PE	Thermo Fisher	Goat IgG

Ig=Immunoglobulin, MHC-II=Major histocompatibility complex class II, FITC=Fluorescein isothiocyanate, APC=Allophycocyanin, PE=Phycoerythrin, BD=Becton Dickinson, WSU=Washington State University

### Cell labeling and flow cytometric analysis

For the immunophenotyping of blood leukocytes [25], separated leukocytes were incubated with mouse monoclonal antibodies to the surface antigens CD4, WC1, and MHC-II and the cell adhesion molecules CD11a, CD11b, and CD18 diluted in MIF buffer for 15 min at 4°C. After incubation, cells were washed 3 times with MIF buffer by centrifugation at 300× *g* for 3 min and discarded the supernatant. Unlabeled primary antibodies were detected using fluorochrome-labeled secondary antibodies, and labeled cells were then analyzed by flow cytometry. A Becton Dickinson FACSCalibur equipped with Cell Quest software (FACSCalibur; Becton Dickinson Biosciences, San Jose, California, USA) was used to collect the data. At least 100,000 cells were collected and analyzed with the software FlowJo version 10 (Flowjo LLC, USA). Negative isotype controls for mouse IgG1, IgG2a, IgG2b (Becton Dickinson), and IgM (Beckman Coulter, CA, USA). were included as part of the study.

### Statistical analysis

Statistical analysis was performed using the software programs Microsoft Excel (2016; Microsoft Corporation, Washington, USA) and Prism V. 5 (GraphPad Software, Inc; California, USA). Results are presented as means±S.E. of the mean (SEM). Differences between means were tested with Student’s t-test and results were considered significant at p<0.05.

## Results

### Forward and side scattering properties of camel calf leukocytes

In the blood of camel calves, neutrophils, eosinophils, lymphocytes, and monocytes showed significantly reduced side scatter (SSC) values (as an indicator for cell granularity) in comparison to adult camels. Only for neutrophils and monocytes, forward scatter (FSC) values (as an indicator for cell size) were lower in calves than in adults (Figure-1a and b).

### Newborn camel calves and adult camels have different leukogram patterns

**Figure-2:**
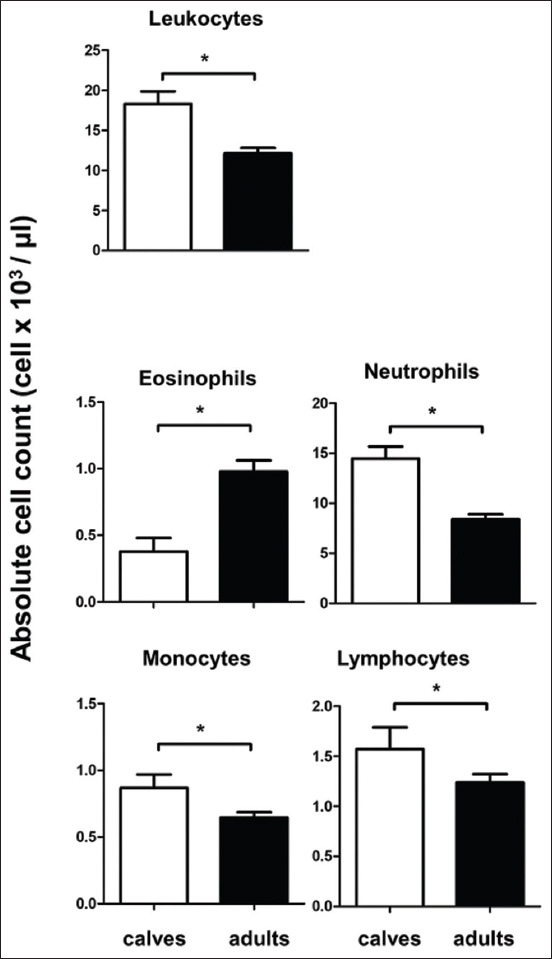
Total and differential cell count of camel blood leukocytes. (a) Total leukocyte count and numbers of neutrophils, eosinophils, lymphocytes, and monocytes in the blood of newborn and adult camels are presented as means±SEM (*p<0.05).

The total cell count of blood leukocytes was significantly higher in camel calves (18.3×10^3^ cell/μL ±1.5) when compared to adult camels (12.6×10^3^ cell/μL ±0.7). The analysis of the absolute count of leukocyte populations revealed higher numbers of neutrophils (14.47×10^3^ cell/μL), lymphocytes (1.57×10^3^ cell/μL), and monocytes (0.87×10^3^ cell/μL) in blood of calves in comparison to the cell numbers of neutrophils (8.7×10^3^ cell/μL), lymphocytes (1.3×10^3^ cell/μL), and monocytes (0.67×10^3^ cell/μL) in blood of adult camels. In opposite to this, adult camels showed significantly higher numbers of eosinophils (1.0×10^3^ cell/μL) in their blood than camel calves (0.38×10^3^ cell/μL) (Figure-2).

### The expression of cell adhesion molecules on leukocyte populations of newborn camel calves

**Figure-3:**
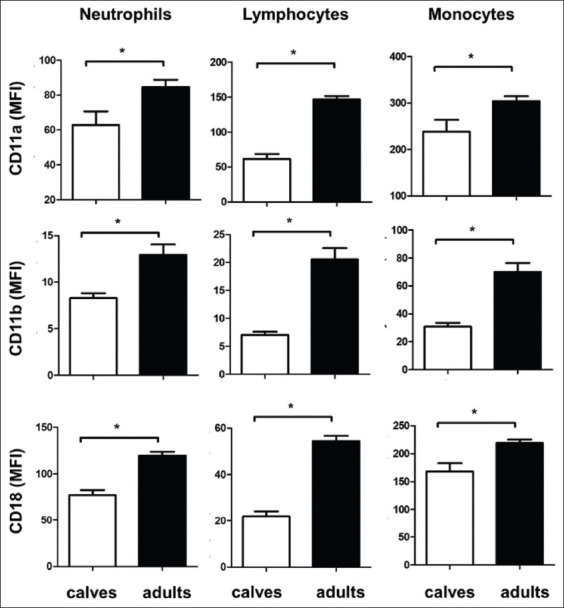
Adhesion molecules expression on leukocyte populations in blood of newborn and adult camels. The differential expression densities of the cell adhesion molecules CD11a, CD11b, and CD18 were estimated as the mean fluorescence intensity of each molecule on the surface of blood neutrophils, lymphocytes, and monocytes. Data for calves and adults were presented graphically (*p<0.05).

For both newborn calves and adult camels, the studied cell adhesion molecules were differently expressed on the main populations of blood leukocytes. All three molecules, CD11a, CD11b, and CD18, were highest expressed on monocytes. Lymphocytes showed higher expression for CD11a and CD11b than granulocytes. CD18 was higher expressed on granulocytes than on lymphocytes. The comparison between camel calves and adult camels revealed significantly lower expression of the cell adhesion molecules, CD11a, CD11b, and CD18 on granulocytes, monocytes, and lymphocytes in comparison to adult camels (Figure-3).

### Lymphocyte subsets in newborn camel calves

**Figure-4:**
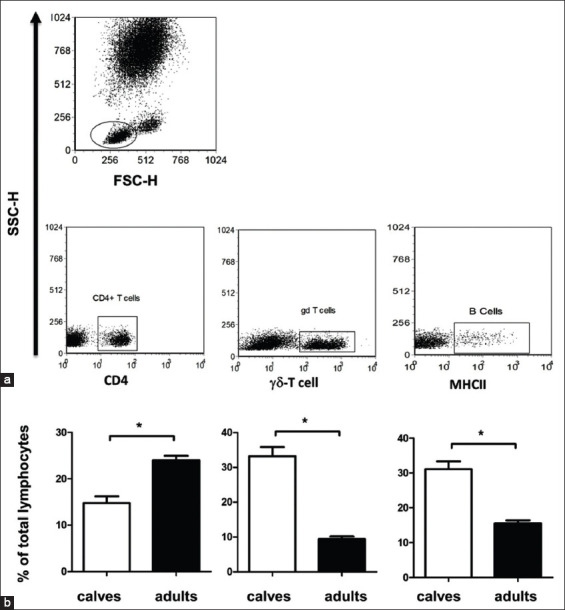
The relative composition of blood lymphocytes in the peripheral blood of newborn and adult camels. (a) After setting a gate on lymphocytes in a SSC/FSC dot plot, the percentages of γ^δ^ T cells, CD4-positive T helper cells, and B cells under total lymphocytes were estimated in separate dot plots according to their staining with the cell-specific marker. (b) Data for newborn and adult camels were presented as mean±SEM (*p<0.05).

In the blood samples of camel calves, there were higher percentages of γδ T cells (33.21%±2.65) and B cells (31.09%±2.27) but lower percentage of T helper cells (14.76%±1.46) in comparison to the percentages of γδ T cells (9.46±0.82), B cells (15.50%±1.10), and T helper cells (24.02±1.06) in blood of adult camels (Figure-4).

## Discussion

For humans, mice, and many other species, different properties of the newborn immune system have been characterized [9,15,27-33].

In the current study, the comparison between camel calves and adult camels revealed higher numbers of total leukocytes and higher numbers of the main leukocyte populations, neutrophils, lymphocytes, and monocytes in calf blood than in adult blood. Studies in other veterinary species [13,14] reported a decrease in lymphocyte count and an increase in neutrophil count with advanced age. The results of the current study, although agrees with the observations regarding the decrease in lymphocyte count with advanced age, seems to be in contrast to earlier studies regarding the increase in the percentage of neutrophil in adults.

Leukocyte count in the peripheral blood is regulated by the balance between leukocyte production in the bone marrow and their migratory activity from blood to tissues [34]. To see whether a defective migration may be responsible for higher leukocyte numbers in calves, the expression of different adhesion molecules on leukocyte populations was compared between calves and adults. In the current study, the expression levels of the cell adhesion molecules, CD11a, CD11b, and CD18, were significantly reduced on calf leukocytes, including neutrophils, lymphocytes, and monocytes, when compared to adult camels. This may indicate retention of leukocytes in calf blood due to impaired adhesion and migration activity of leukocytes, which could be responsible for higher numbers of these cells in calf blood. For human, age-related changes in the expression pattern of leukocyte adhesion molecules have been reported in different studies [9,22-24].

It has been reported that the count of eosinophils, which play a major role in parasitic immunity, increases with advanced age due to increased parasitic infestations [35]. Although not proven in the current study, the higher numbers of eosinophils in adult camels may be due to higher parasitic manifestation in these animals than in newborns [36].

The comparative analysis of the relative composition of camel blood lymphocytes revealed the presence of higher percentages of γδ T cells and B cells in blood of camel calves in comparison to adults. In contrast to this, camel calves showed reduced percentages of CD4-positive T helper cells than adult camels. Animal’s age has a strong impact on phenotype and composition of blood lymphocytes [9]. Studies on the immune system of bovine calves have indicated a rise in absolute numbers of lymphocytes, mainly the numbers of B cells and CD4+ T cells, in calves up to 9-11 weeks after birth [15]. However, other studies have reported stable percentages of helper T cells, cytotoxic T cells, and γδ T cells during the first 10-12 weeks after birth [37].

Flow cytometric analysis of SSC and FSC, which are characteristic for cell granularity and cell size, respectively, has been widely used for the assessment of cell activation status [38,39]. In the present study, the reduced SSC and FSC values for different leukocyte populations of camel calves indicate reduced activation status of calf leukocytes, which may contribute to reduced functionality of the newborn immune system in camels.

## Conclusion

This is the first study on the distribution and immunophenotype of leukocyte subpopulations in the peripheral blood of newborn dromedary camels. With higher total leukocyte numbers, higher numbers of neutrophils, monocytes, and lymphocytes, and lower numbers of eosinophils, newborn calves show a different leukogram in comparison to adult camels. The fractions of B cells and γδ T cells were also elevated in calves blood, whereas the fraction of T helper cells was reduced in comparison to adult animals. The observed rise in many leukocyte populations in calf blood may be due to reduced expression of the cell adhesion molecules, CD11a, CD11b, and CD18 on the surface of calf leukocytes.

## Authors’ Contributions

JH and MMG conceived and designed the study. MMG collected the samples from the animals and prepared the samples for flow cytometry. JH and MMG analyzed the labeled cells by flow cytometry. MMG, JH, and AIAA wrote the final manuscript. All authors have read and approved the final manuscript.
